# Analgesia Nociception Index-Guided Remifentanil versus Standard Care during Propofol Anesthesia: A Randomized Controlled Trial

**DOI:** 10.3390/jcm11020333

**Published:** 2022-01-11

**Authors:** Nada Sabourdin, Julien Burey, Sophie Tuffet, Anne Thomin, Alexandra Rousseau, Mossab Al-Hawari, Clementine Taconet, Nicolas Louvet, Isabelle Constant

**Affiliations:** 1 Département d’Anesthésie-Réanimation, Hopital Trousseau, GRC 29, DMU DREAM, Sorbonne Université, AP-HP, 75012 Paris, France; mossab.alhawari@aphp.fr (M.A.-H.); nicolas.louvet@aphp.fr (N.L.); isabelle.constant@aphp.fr (I.C.); 2 EA 7323: Pharmacologie et Evaluation des Thérapeutiques chez L’enfant et la Femme Enceinte, Université de Paris, 75006 Paris, France; 3 Département d’Anesthésie-Réanimation, Hopital Tenon, GRC 29, DMU DREAM, Sorbonne Université, AP-HP, 75020 Paris, France; julien.burey@aphp.fr (J.B.); clementine.taconet@aphp.fr (C.T.); 4 Department of Clinical Pharmacology and Clinical Research Platform of the East of Paris (URC-CRC-CRB), Hôpital St Antoine, AP-HP, 75012 Paris, France; sophie.tuffetsouloumiac@aphp.fr (S.T.); alexandra.rousseau@aphp.fr (A.R.); 5 Département de Gynécologie et Obstétrique, Hopital Trousseau, FHU PREMA, Sorbonne Université, AP-HP, 75012 Paris, France; anne.thomin@aphp.fr

**Keywords:** analgesia nociception index, ANI, monitoring, nociception, remifentanil, intravenous anesthesia

## Abstract

The clinical benefits to be expected from intraoperative nociception monitors are currently under investigation. Among these devices, the Analgesia Nociception-Index (ANI) has shown promising results under sevoflurane anesthesia. Our study investigated ANI-guided remifentanil administration under propofol anesthesia. We hypothesized that ANI guidance would result in reduced remifentanil consumption compared with standard management. This prospective, randomized, controlled, single-blinded, bi-centric study included women undergoing elective gynecologic surgery under target-controlled infusion of propofol and remifentanil. Patients were randomly assigned to an ANI or Standard group. In the ANI group, remifentanil target concentration was adjusted by 0.5 ng mL^−1^ steps every 5 min according to the ANI value. In the Standard group, remifentanil was managed according to standard practice. Our primary objective was to compare remifentanil consumption between the groups. Our secondary objectives were to compare the quality of anesthesia, postoperative analgesia and the incidence of chronic pain. Eighty patients were included. Remifentanil consumption was lower in the ANI group: 4.4 (3.3; 5.7) vs. 5.8 (4.9; 7.1) µg kg^−1^ h^−1^ (difference = −1.4 (95% CI, −2.6 to −0.2), *p* = 0.0026). Propofol consumption was not different between the groups. Postoperative pain scores were low in both groups. There was no difference in morphine consumption 24 h after surgery. The proportion of patients reporting pain 3 months after surgery was 18.8% in the ANI group and 30.8% in the Standard group (difference = −12.0 (95% CI, −32.2 to 9.2)). ANI guidance resulted in lower remifentanil consumption compared with standard practice under propofol anesthesia. There was no difference in short- or long-term postoperative analgesia.

## 1. Introduction

The monitoring of nociception has made substantial progress in the last decade. Several devices have been validated as monitoring tools for intraoperative nociception.

Among these devices, the Analgesia Nociception Index (ANI, Mdoloris Medical Systems, Loos, France) has the particularity of assessing more specifically the parasympathetic component of the autonomic response. Using the electrocardiogram signal as an input, it provides a continuous, normalized and automated index, comprised between 0 and 100, derived from the high-frequency component of heart rate variability. In anesthetized patients, the ANI decreases when the level of analgesia does not compensate the intensity of the nociceptive stimulation [1,2,3]. The amplitude of this ANI decrease is positively related to the intensity of the stimulation, and inversely correlated to the amount of opioids [4,5,6,7]. Based on the first published studies, the manufacturer suggests that an ANI of between 50 and 70 may correspond to adequate antinociception.

The final goal of the ANI, as for all nociception monitors, is to improve patients’ outcomes. Individually adjusting opioid administration according to this pharmacodynamic biofeedback throughout the surgical procedure is expected to limit opioid underdosage and overdosage. This might improve hemodynamic stability, prevent opioid tolerance and hyperalgesia, and also decrease the incidence of opioid-related side effects.

Two studies, performed under sevoflurane anesthesia, have shown encouraging results regarding ANI-guided intraoperative opioid administration, with either intraoperative [8] or early postoperative [9] benefits. Conversely, another study failed to demonstrate any advantage of ANI guidance over standard practice [10]. These studies were included in a recent meta-analysis, concluding that under sevoflurane anesthesia, the difference between ANI-guided analgesia and standard care was not significant [11]. This meta-analysis, along with recent literature reviews [12,13], considered the available data insufficient to draw definitive conclusions, and called for additional trials. In particular, there were no published data about ANI guidance of intraoperative opioids under propofol anesthesia.

Propofol and sevoflurane both depress global autonomic nervous activity, but their specific effects on the sympathetic and parasympathetic components of the autonomous nervous system are different [14,15]. The ANI data obtained under sevoflurane cannot be directly extrapolated to propofol anesthesia.

We designed this prospective randomized controlled trial to assess the potential benefits of ANI-guided remifentanil administration in patients under propofol anesthesia. We hypothesized that ANI guidance would result in lower intraoperative remifentanil consumption, which was our main outcome measure. We also studied postoperative morphine requirements, pain and opioid-related side-effects.

## 2. Materials and Methods

This was a prospective, randomized, controlled, single-blinded study conducted in two centers: Armand Trousseau Academic Hospital (Paris, France) and Tenon Academic Hospital (Paris, France). The trial was approved by the Ethics Committee for the Protection of Persons (CPP Sud-Ouest et Outre-Mer, Number 1-18-38, 9 July 2018), and registered at clinicaltrials.gov (NCT03498820) before the first inclusion. Written informed consent was obtained from each patient prior to enrollment.

### 2.1. Population

We included 18–60 year-old women, ASA status I-II, scheduled for a gynecologic surgery under general anesthesia, with an expected procedural duration of at least 60 min. We did not include obese patients, patients with chronic pain, patients with a history of substance abuse or psychiatric disease, patients with cardiac arrhythmia or a pacemaker or patients with a neurologic or metabolic disease.

### 2.2. Study Design

#### 2.2.1. Randomization

After inclusion, patients were randomly assigned to ANI group or Standard group, using a secure web-response system (CleanWEB Telemedicine Technologies SAS, Boulogne-Billancourt, France). Randomization (1:1 ratio) was stratified on center and block balanced (different widths of blocks, not communicated to investigators). Randomization was prepared by an independent statistician. The patients were blinded to their group allocation.

#### 2.2.2. Anesthesia

Upon arrival in the operating room, standard monitoring was initiated: electrocardiogram, non-invasive blood pressure, oxygen saturation, bispectral index (BIS; Covidien, Dublin, Ireland), and temperature. In addition, ANI monitoring was used for all patients, with the thoracic cutaneous electrode placed according to the manufacturer’s recommendation. For this study, we only considered “instantaneous ANI”, averaged over one minute, with a new value displayed every second.

*Induction:* Anesthesia was induced by effect-site target-controlled infusion (Base Primea; Fresenius-Kabi, Bad Homburg, Germany) of propofol and remifentanil. The initial effect-site estimated target concentrations (Ce) were set at 6 mg mL^−1^ for propofol (Schnider model), and 4 ng mL^−1^ for remifentanil (Minto model). Patients received atracurium 0.5 mg kg^−1^ for intubation. Then, remifentanil Ce was set at 2 ng mL^−1^. Ventilation was adjusted to maintain end-tidal carbon dioxide between 35 and 45 mmHg.

*Maintenance:* Propofol was adjusted to target a BIS of (40–60). If the train-of-four induced more than one response before the beginning of wound closure, patients received atracurium 0.25 mg kg^−1^. Remifentanil management depended on the group:

- In the Standard group, intraoperative remifentanil management was left to the discretion of the anesthesiologist in charge of the patient, according to their usual practice. In both institutions, ANI monitoring was not part of standard practice, so in this group, the anesthesiologist was blinded to the ANI.
- In the ANI group, intraoperative remifentanil was guided by the ANI value, and could be modified every 5 min. If ANI was <50, remifentanil Ce was increased by 0.5 ng mL^−1^. If ANI was >70, remifentanil Ce was decreased by 0.5 ng mL^−1^. If ANI was between 50 and 70, no changes were made. The minimal remifentanil Ce allowed in the protocol was 1 ng mL^−1^. Several safety items regarding the management of hemodynamic changes were added to our algorithm: if SBP was >130 mmHg on two consecutive measurements, nicardipine 1 mg kg^−1^ was administered. Conversely, if SBP was <80 mmHg, patients received 500 mL of crystalloids. If SBP remained low, ephedrine was administered (3 mg boluses) until blood pressure was restored. Finally, if heart rate (HR) decreased below 45 bpm, patients received atropine (1 mg). Atropine or ephedrine administration resulted in the discontinuation of the ANI-guidance algorithm: patients were managed as in standard practice for the rest of the procedure.

*Emergence:* Patients received 15 mg kg^−1^ of paracetamol (maximum 1 g) and 0.05 mg kg^−1^ of morphine 30 min before the end of the procedure. Remifentanil was discontinued at the completion of wound closure. After wound closure, a Transversus Abdominis Plane block (laparotomy) or port-site local anesthetic infiltration (laparoscopy) was performed with ropivacaine (2 mg ml^−1^). Patients were then extubated and transferred to the post-anesthesia care unit (PACU).

#### 2.2.3. Postoperative Period

In PACU, morphine was titrated with 1 mg boluses every 3 min until the visual analog scale (VAS) score was <4/10. Then, morphine was managed by a standardized patient-controlled analgesia device (1 mg boluses, 6 min refractory period). Patients received 15 mg kg^−1^ of scheduled intravenous paracetamol (maximum 1 g) every 6 h.

If VAS score was >3/10 despite morphine and paracetamol, patients received nefopam 20 mg IV every 6 h as necessary. If VAS score remained >4/10, patients received ketoprofen 50 mg IV every 8 h as needed.

### 2.3. Data Collection

Intraoperative data: Baseline HR and SBP were obtained just before skin incision. Then, HR, SBP, BIS, propofol Ce, remifentanil Ce and ANI were collected every five min from skin incision until wound closure. For example, during a 3-h procedure, 36 sets of data were obtained. At the end of the procedure, total doses of remifentanil and propofol were recorded, as well as the number of atropine, ephedrine and nicardipine boluses. Surgeons were also asked to rate their overall satisfaction on a 4-points scale (0 = not satisfied at all, 3 = very satisfied). All intraoperative data were collected by an investigator dedicated to the protocol. This investigator could not be blinded to the patient’s group.

Postoperative data: HR, SBP, SpO_2_ and pain score (Visual Analogue Scale, VAS) were collected every 15 min in PACU. Then, HR, SBP, VAS, morphine consumption as well as opioids-related side effects were collected 2, 4, 6, 12 and 24 h after the end of surgery by nurses, blinded to the group allocation. On postoperative day 1, an investigator, unaware of the group, interviewed the patients using a standard set of questions to detect possible intraoperative awareness. Patients were also asked to rate their overall satisfaction on a 4-points scale. Finally, a phone interview was scheduled with each patient 3 months after the procedure. The patients were asked if they still felt pain on the surgical site, and they could answer “yes” or “no”. The interviews were conducted by an investigator unaware of the group allocation.

### 2.4. Aims and Outcome Measures

Our primary objective was to compare intraoperative remifentanil consumption between ANI and Standard groups. The main outcome measure was the total dose of remifentanil (μg kg^−1^ h^−1^).

Our secondary objectives were to compare between the groups:

- The quality of anesthesia: the related outcome measures were the total dose of propofol, the number of changes in remifentanil Ce, duration of anesthesia, time to emergence (between remifentanil discontinuation and extubation), potential awareness and surgeon’s satisfaction.
- Postoperative analgesia: the related outcome measures were postoperative cumulated morphine consumption 12 and 24 h after surgery, the proportion of patients requiring nefopam or ketoprofen, VAS scores, incidence of nausea, vomiting, urinary retention, respiratory depression requiring oxygen administration and patient’s satisfaction.
- Chronic pain: presence of pain 3 months after the procedure.

### 2.5. Statistical Analysis

Assuming a mean intraoperative remifentanil consumption of 7.8 ± 1.9 μg kg^−1^ h^−1^ in the Standard group [16], 80 randomized patients were needed to achieve 90% power to detect a relative decrease of 20% in remifentanil consumption in the ANI group, considering a non-parametric test with a two-sided alpha of 5% and an expected dropout rate of 10%.

Baseline characteristics, preoperative postoperative data were reported using frequency (%) for categorical variables and mean ± standard deviation (SD) or median (interquartile range (IQR)) for quantitative variables, depending on their distribution. The analysis was performed according to intent-to-treat (ITT) principle, among the as-randomized patients who underwent the surgical procedure. Total dose of remifentanil was compared using Wilcoxon Mann–Whitney test. Missing data were replaced by the maximum value observed (worst-case scenario). Sensitivity analyses were performed among the as-randomized population with available data and on the per-protocol population. Per-protocol population was defined as as-randomized population excluding patients with a missing primary outcome value, non-respected eligibility criteria, patients receiving ephedrine or atropine or with a procedure duration shorter than 1 h.

To avoid multiple testing, secondary outcomes were compared using a confidence interval approach. Differences (ANI minus Standard group) of means or medians or proportions were calculated with their 95% CIs evaluated using normal approximation, Mood method and Exact method, respectively. In each group, 95% CIs were estimated by normal approximation for means, by distribution-free method for medians and by Exact method for proportions. Post hoc analyses included the number of patients requiring ephedrine, atropine and nicardipine and descriptive plots of remifentanil consumption over surgical time.

Analyses were performed with SAS software version 9·4 (SAS Institute Inc., Cary, NC, USA), except for 95% CI differences estimated with R Studio version 1.2.5019 (R: A Language and Environment for Statistical Computing, R Core Team, R Foundation for Statistical Computing, Vienna, Austria, https://www.R-project.org). All tests were two-sided and a *p* value of less than 0.05 indicated statistical significance.

## 3. Results

This section may be divided by subheadings. It should provide a concise and precise description of the experimental results, their interpretation, as well as the experimental conclusions that can be drawn.

### 3.1. Population

**Figure 1:**
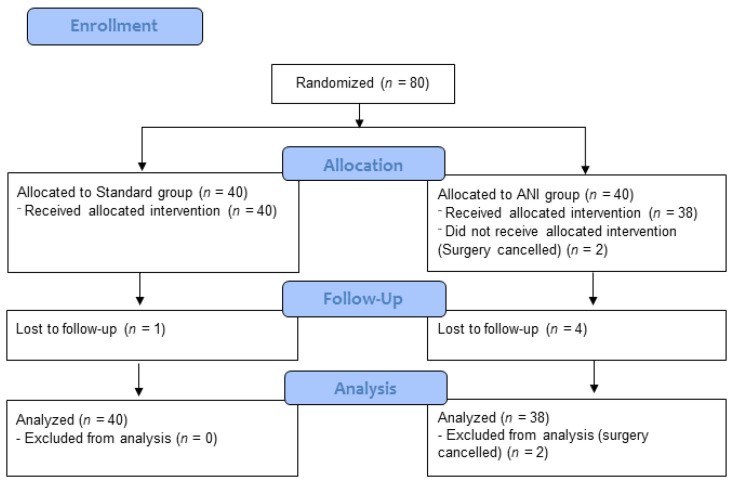
CONSORT flow diagram.

Eighty patients were included between November 2018 and December 2019. Among them, 78 underwent the surgical procedure (hysterectomy, *n* = 35; myomectomy, *n* = 23; adnexectomy, *n* = 7, other, *n* = 13; two procedures were cancelled) and were included in the ITT analysis. ANI guidance was interrupted in three patients of the ANI group because they received ephedrine or atropine. The flowchart is provided in Figure 1; patients’ characteristics are provided in Table 1. In the 78 patients who underwent surgery, a total of 1864 intraoperative datasets were analyzed between skin incision and wound closure.

### 3.2. Remifentanil

**Figure 2:**
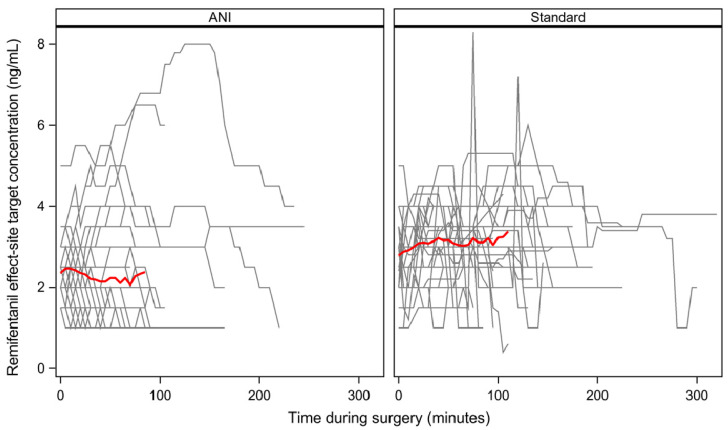
Individual evolution of remifentanil effect-site target concentration in the ANI (38 lines; **left**) and Standard group (40 lines; **right**). Each line represents a patient. Red lines represent the means.

Median remifentanil consumption was lower in the ANI group: 4.4 (3.3; 5.7) vs. 5.8 (4.9; 7.1) µg kg^−1^ h^−1^ (difference = −1.4 (95% CI, −2.6 to −0.2), *p* = 0.0026). These results were consistent with the sensitivity analyses restricted to patients with available data (*n* = 74, difference = −1.4 (95% CI, −2.8 to −0.7), *p* < 0.001) and on the per-protocol population (*n* = 71, difference = −2.0 (95% CI, −2.9 to −0.9), *p* < 0.001). Individual trajectory plots of remifentanil Ce during surgery are provided in Figure 2.

### 3.3. Quality of Anesthesia 

Propofol consumption and time to emergence were not different between the groups (Table 2). Duration of anesthesia was shorter in the ANI group. Overall, four patients received atropine, three received ephedrine, and one received nicardipine.

**Figure 3:**
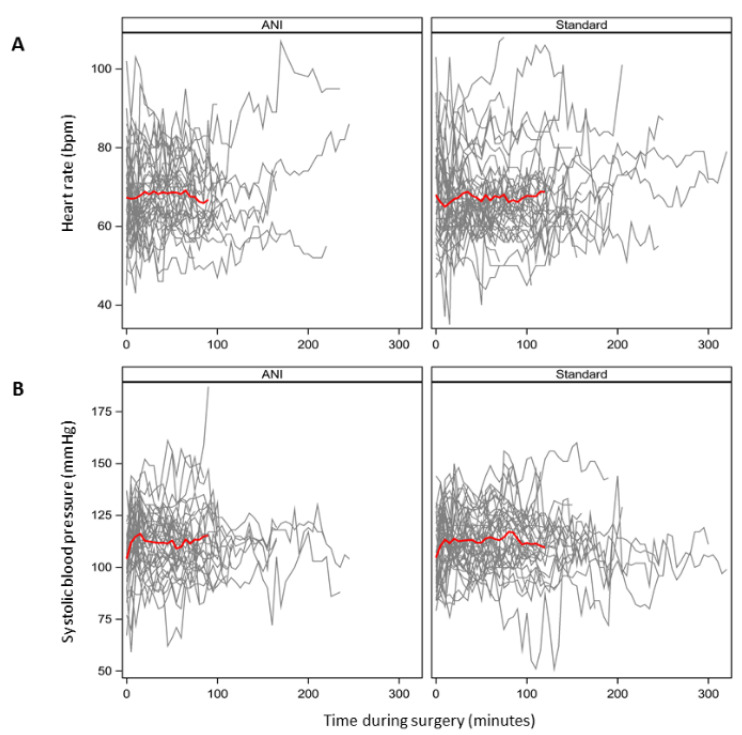
Individual evolution of heart rate (**A**) and systolic blood pressure (**B**) in the ANI group (38 lines; **left**) and Standard group (40 lines; **right**). Each line represents a patient. Red lines represent the means.

Individual evolution of heart rate and systolic blood pressure are provided in Figure 3. Surgeons were more frequently “very satisfied” in the ANI group: 89.5% vs. 69.2% in the Standard group (difference = 20.2% (95% CI, 1.7% to 39.2%)). No awareness was suspected.

### 3.4. Postoperative Analgesia 

**Figure 4:**
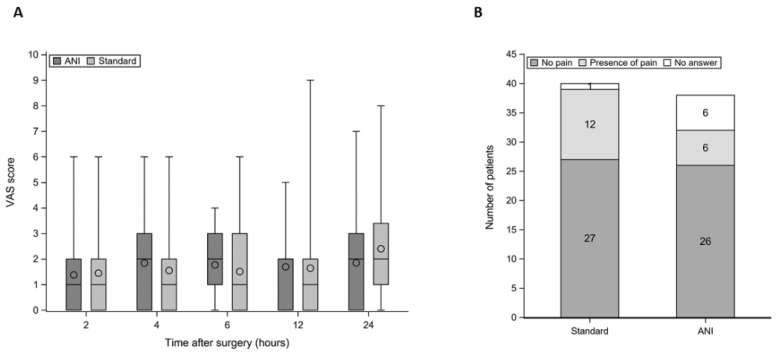
Postoperative pain with (**A**) acute pain during the first 24 h, by visual analog scale (VAS) in the ANI and Standard groups. Mean is represented by circle and median by the horizontal line inside the box; and (**B**) chronic pain at the surgical site 3 months after the procedure (phone survey). Light gray indicates number of patients who reported persistent pain; dark gray indicates number of patients who reported no persistent pain; white indicates number of patients who did not answer our phone calls.

There was no difference between the groups in cumulated morphine consumption 12 or 24 h after surgery (Table 2). The incidence of opioids-related side effects was comparable between the groups. The proportion of patients requiring nefopam or ketoprofen for rescue analgesia was similar between the groups. Postoperative VAS scores were low in both groups (Figure 4). The proportion of patients who were “very satisfied” was higher in the ANI group: 83.3% vs. 38.5% in the Standard group (difference = 44.9% (95% CI, 20.8% to 63.8%)).

### 3.5. Chronic Pain

Five patients were lost to follow-up after hospital discharge (did not answer our phone calls after five attempts) and data were unavailable for two other patients. Thus, 71 answers were analyzed (Figure 4). The proportion of patients reporting pain 3 months after surgery was 18.8% in the ANI group versus 30.8% in the Standard group (difference = −12.0 (95% CI, −32.2% to 9.2%)).

## 4. Discussion

Under propofol anesthesia, ANI guidance of intraoperative remifentanil resulted in a significant decrease in total remifentanil consumption, while maintaining a stable anesthesia. This decrease was not associated with modifications in postoperative morphine requirements, or opioids-related side-effects. Fewer patients in the ANI group reported chronic pain 3 months after surgery, but this difference was not significant.

Under volatile anesthetic agents, the ANI has been used to guide intraoperative opioids in different settings, and with various results. In bariatric and breast surgery, ANI guidance resulted in a decrease in intraoperative opioid consumption [8,17]. No postoperative benefits were evidenced in these studies. Conversely, in spine surgery, ANI guidance did not result in a difference in intraoperative opioid consumption [9,18], but could be associated with benefits in the first 90 min after emergence: lower pain scores, less postoperative morphine, less nausea. Finally, during laparoscopic cholecystectomy, intraoperative opioid dosage was not reduced by ANI guidance, and no postoperative benefits were evidenced.

These results, although indisputably conflicting, should be interpreted with caution because of the heterogeneity in the anesthetic agents, surgical procedures and populations. Several devices have been validated as quantitative nociception monitors [7,19]. Pupillometry [16], Surgical Pleth index [20] and Nociception Level [21] have demonstrated an intraoperative opioid-sparing effect under propofol–remifentanil anesthesia: our study demonstrates that ANI has the same ability in our experimental conditions. However, as is the case with the ANI, there are also negative reports with each of these monitors used in different anesthetic conditions [22,23,24]. The potential benefits of nociception monitoring do not only depend on the choice of the monitoring device; they also depend on the global anesthetic strategy and surgical context, the thresholds of intervention and the target population. The current issue may now be to determine the best way to use each of the validated nociception monitors, in order to assess “if” and “how” they can be useful for the patients [12]. Our results, along with the other published data, suggest that ANI monitoring could prove more useful under propofol than under volatile agents, with short-acting opioids such as remifentanil, during long rather than brief surgical procedures.

The size of the opioid-sparing effect we reported was consistent, with a difference of approximately 30% between the groups. In a previous study, our team investigated the benefits associated with pupillometry-guided remifentanil in a very similar context: the same population, surgical procedures, anesthetic agents, and frequency and amplitude of remifentanil steps [16]. We had evidenced a decrease in remifentanil consumption of approximately 50% in the pupillometry-guided group.

However, interestingly, median remifentanil consumption in the former pupillometry group (3.8 μg kg^−1^ h^−1^) was of the same order as the dose received in our ANI group. Propofol consumptions in both studies were also comparable. Thus, using either the ANI or pupillometry in similar populations and similar surgical contexts led to relatively close remifentanil consumptions.

In contrast, median remifentanil consumption in the Standard group of our current study was well below the one reported in the Standard group of the pupillometry study (7.9 μg kg^−1^ h^−1^). Our relatively low standard remifentanil consumption might reflect the fact that, at least in our center, standard practice has changed since we published the pupillometry study.

ANI guidance did not result in more frequent adaptations of remifentanil Ce. The decrease in remifentanil consumption might be explained by differences in the timing, amplitude and direction of the Ce changes (Figure 2).

There were no substantial intraoperative hemodynamic differences between the groups. Only one patient in the ANI group received nicardipine (single bolus), and very few patients received atropine or ephedrine in both groups. In our setting, ANI guidance allowed for us to maintain a stable anesthesia while reducing opioid consumption.

Remifentanil has been associated with postoperative hyperalgesia. In our former study [16], pupillometry led to a moderate decrease in postoperative morphine. In contrast, in this study, we failed to demonstrate any association between the lower remifentanil consumption in the ANI group and postoperative morphine consumption. This result might have several explanations. The monitor-related remifentanil decrease was less marked in this study: the amplitude of the difference might not have been sufficient to induce a significant postoperative opioid-sparing effect. In addition, the amount of remifentanil administered in the Standard group might have been too low to induce hyperalgesia.

Hyperalgesia has been associated with the development of chronic pain. The proportion of patients who reported persistent pain 3 months after surgery was lower in the ANI group. However, the width and overlap of the confidence intervals did not allow for us to conclude a difference between the groups. Our study was not designed to answer this question, and might have lacked the necessary power to detect a difference in chronic pain incidence. In addition, we did not use a validated chronic pain questionnaire during the telephone interview.

Our study has several other limitations. Our anesthetic protocol did not include anti-hyperalgesic medications such as ketamine, or other adjuvants such as alpha-2 agonists or lidocaine. We discontinued ANI guidance when atropine or ephedrine was administered. These agents are expected to modify the ANI, but the amplitude and duration of their effect on the index have not yet been precisely described. Whether ANI guidance still makes a difference when these agents are used remains to be demonstrated. In addition, our study was performed in a very specific population: we did not include elderly, diabetic or obese patients or patients with chronic pain or arrhythmias. Thus, our patients do not represent the usual population managed by anesthesiologists on a daily basis. Whether our results can be extrapolated to a wider population also remains to be demonstrated. Finally, because it is our institutional standard practice, we used two different regional anesthesia techniques, depending on the surgery (TAP-block for laparotomies, wound infiltration for laparoscopies); although these techniques are described as equivalent in gynecological surgery [25], this might have affected the amount of analgesia required in the early postoperative period. However, as the proportion of laparoscopies/laparotomies was equivalent in both groups (Table 1), it is unlikely that our results were influenced by the techniques of regional anesthesia.

In conclusion, ANI-guided remifentanil administration resulted in lower remifentanil consumption compared with standard practice, while maintaining a stable anesthesia in healthy women undergoing major gynecological surgery under propofol–remifentanil TCI. This technique was not associated with peri-operative complications, and there was no difference in postoperative outcomes. ANI, along with other nociception monitors, should be further investigated under different experimental conditions to optimize the potential clinical benefits they may bring to patients.

## Figures and Tables

**Table 1:** Patient characteristics. Baseline measurements were made under general anesthesia, just before surgical incision. Data are presented as mean ± standard deviation.

	ANI (*n* = 38)	Standard (*n* = 40)
Age (years)	39 ± 10	40 ± 7
Weight (kg)	68 ± 10	68 ± 11
Body Mass Index (kg/m^2^)	24.9 ± 3.4	24.9 ± 3.2
Baseline SBP (mmHg)	100 ± 14	104 ± 14
Baseline HR (bpm)	70 ± 11	70 ± 12
Laparoscopy (*n*)	19	19
Laparotomy (*n*)	19	21

ANI: Analgesia Nociception Index, SBP: systolic blood pressure, HR: heart rate.

**Table 2:** Quality of anesthesia and postoperative analgesia. Data are presented as Number (*n*), mean ± SD or median (95% confidence interval).

	ANI (*n* = 38)	Standard (*n* = 40)
**Per-operative data**		
Propofol consumption (mg kg^−1^ h^−1^)	8.4 ± 1.7	8.1 ± 1.6
Changes in Remifentanil Ce (Total number)	5 (4; 7)	5.0 (2; 7)
Time to emergence (min)	13.5 (7.0; 21.0)	15.5 (10.0; 26.0)
Duration of anesthesia (min)	133 (114; 161)	168 (135; 212)
Patients requiring atropine (*n*)	2	2
Patients requiring ephedrine (*n*)	1	2
Patients requiring nicardipine (*n*)	1	0
**Postoperative data**		
12 h cumulative morphine (mg kg^−1^)	0.22 (0.17; 0.25)	0.23 (0.19; 0.32)
24 h cumulative morphine (mg kg^−1^)	0.25 (0.21; 0.31)	0.29 (0.21; 0.39)
Proportion of patients requiring nefopam (%)	73.5% (55.6%; 87.1%)	75.7% (58.8%; 88.2%)
Proportion of patients requiring ketoprofen (%)	35.3% (19.7%; 53.5%)	19.4% (8.2%; 36.0%)
Nausea/Vomiting (*n* (%))	8 (25%)	8 (21.6%)
Itching (*n* (%))	1 (3.1%)	0
Urinary retention (*n* (%))	0	2 (5.1%)

ANI: Analgesia Nociception Index.

## Data Availability

Data are available on request from the corresponding author.
